# Seasonal fluctuations in body weight during growth of Thoroughbred racehorses during their athletic career

**DOI:** 10.1186/s12917-017-1184-3

**Published:** 2017-08-18

**Authors:** Yuji Takahashi, Toshiyuki Takahashi

**Affiliations:** Sports Science Division, Equine Research Institute, Japan Racing Association, 1400-4, Shiba, Shimotsuke, Tochigi 329-0412 Japan

**Keywords:** Body weight, Growth, Racehorse, Seasonal change, Sex difference

## Abstract

**Background:**

Domesticated horses adapt to environmental conditions through seasonal fluctuations in their metabolic rate. The seasonal change of metabolic rates of domesticated horses in pastures is documented. However, there are few investigations on seasonal body weight change of domesticated horses housed in stables, which are provided constant energy intake throughout the year. Both seasonal changes and gain in body weight of racehorses during their athletic career is known to a lesser extent because their body weight are not measured in most countries. Here, we used a seasonal-trend decomposition method to conduct a time series analysis of body weight of Thoroughbred racehorses participating in flat races held by the Japan Racing Association from 1 January 2002 to 31 December 2014.

**Results:**

We acquired 640,431 body weight measurements for race starts and included 632,540 of these in the time series analysis. Based on seasonal component analysis, the body weight of male and gelding horses peaked in autumn and winter and reached its nadir in summer. In contrast, the body weight of female horses peaked in autumn and reached the nadir in spring. Based on trend component analysis, most of the increase in body weight was observed when all sexes approached 5 years of age. The slope of the body weight gain was smaller after that, and an approximately 30 kg gain was observed during their careers.

**Conclusion:**

These results indicate that the body weight of a Thoroughbred racehorse fluctuates seasonally, and that there may be sex differences in energy balance mechanisms. Moreover, the present results suggest that the physiological development of Thoroughbred racehorses is completed just before they reach 5 years of age.

## Background

Seasonal changes in food availability and quality are inevitable. Because of changes in forage in the wild, the body weight of a wild herbivore varies seasonally [1, 2]. Przewalski horses, an ancestor of domesticated horses, which live in a semi-natural environment, exhibit annual fluctuations in body weight that peak in autumn and reach the nadir in spring [3]. These fluctuations are associated with energy quality and quantity derived from dry matter intake or with dietary composition [4]. Further, the energy expenditure of Przewalski horses is low in winter to adjust to food shortages and high in spring owing to pregnancy [5]. Energy balance varies with seasonal fluctuations depending on the relationship between energy intake and expenditure [6]. Therefore, body weight increases during an energetically abundant season and decreases during an energetically deficient season [3, 6].

In contrast to wild animals, the amount of forage available to domesticated animals is not affected by the season. However, recent studies reveal that domesticated mammals can change their energy expenditure depending on seasonal environmental changes such as temperature or photoperiod [7–9]. For example, the metabolic rate of a Shetland pony mare is high in summer and low in winter, according to climate change [8]. This seasonal metabolic change suggests that the body weight of a domesticated horse can change with seasonal fluctuations that are high in winter and low in summer, despite constant feeding. Although some studies investigated seasonal changes of the body weight of domesticated horses, including racehorses [7, 8, 10–12], most of the horses were kept on pastures with amounts of forage that varied between seasons. Therefore, little is known about seasonal changes of body weight that occur when energy intake is constant throughout the year.

In the equine industry, many investigations report growth curves from birth to the yearling stage [13–16] as well as the change in the body weight of mares [10, 17]. These studies show how horses grow from birth to approximately 700 days of age and how body weight changes during reproduction. This information would be helpful for providing appropriate nutrition during these times to prevent diseases such as laminitis or metabolic syndrome [3, 18, 19].

During a horse’s athletic career, controlling body weight is considered important, because body weight can be associated with the risk of injury [20] or performance level [21]. From the perspective of skeletal development, we can expect an increase in body weight until the horse approaches 5 years of age, the time of the latest epiphysis closure [22]. However, few published investigations measured body weight changes over several years during a horse’s athletic career [11] because body weight is not measured in most countries, except for some Asian countries, including Japan.

In Japan, there is no off-season for racing and about 65 flat races, from Maiden Races up to Group One (the highest class), are held every week throughout the year. The horses are all given an official birthday of January 1st to keep the age groups easily defined for race conditions; horses aged 2 years are allowed to debut in June, although most horses, which cannot win a Maiden Race until the end of September aged 3 years, are forced to retire due to the lack of their prize money. The Japan Racing Association (JRA) records body weight data for all horses participating in races. Therefore, the analysis of these data provides an opportunity to improve our understanding of the growth and seasonal body weight changes of racehorses.

Although Cho et al. investigated the average body weight of Thoroughbred horses of all sexes according to month and age separately [11], it is difficult to identify the component that determines body weight change during growth. This is because body weight during growth can be affected by seasonal effects, as described above, or by growth, genetic effects, nutrition, and so on. Therefore, dividing body weight data into seasonal and growth components is valuable and could have important implications for planning the nutritional management of athletic horses to prevent injury, increase performance, or both.

To investigate how the body weight of Thoroughbred racehorses changes during their careers, we conducted a time-series analysis by dividing the data into seasonal, trend and remainder components. We hypothesized that body weight changes between seasons and increases just before the age of 5 years.

## Methods

We acquired body weights of racehorses at flat races held by the JRA from 1 January 2002 to 31 December 2014 from the JRA’s official database. Permission to use this dataset for the present study was given by the Equine Research Institute of JRA. Each year, approximately 3400 flat races are held by the JRA, including 48,000 race starts. The JRA operates racecourses at Sapporo, Hakodate, Fukushima, Niigata, Tokyo, Nakayama, Chukyo, Kyoto, Hanshin and Kokura, which range in latitude from N 34° to N 43°.

In the present study, all horses were stabled at the Miho (N 36, E 140°) or the Ritto (N 35°, E 136) Training Centre in Japan for at least 10 days before races. Except during training, the horses were housed individually in stalls (2.8 × 4.0 m) under natural photo-thermoperiod conditions and at the ambient temperature. Training was typically performed for 90 to 120 min each day, 6 days a week, and workout training was performed once or twice each week; this is classified as very heavy work according to the National Research Council [23]. Stable staff controlled the care of the racehorses. However, we recommended to staff that the nutritional energy requirements of the horses should be calculated according to the following equation, and stable staff should feed the horses accordingly. The equation, which is for horses undergoing very heavy work, is as follows [23]:

$$ \mathrm{Digestible}\  \mathrm{energy}\ \left(\mathrm{Mcal}/\mathrm{day}\right)=\left(0.0363\times \mathrm{body}\  \mathrm{weight}\right)\times 1.9 $$

The horses received their daily quantity of food as either two or three meals and water was available ad libitum in each stall. Generally, the dietary treatment consisted of mixed feed at 0.8%–1.2% body weight, oats at 0.5%–1.0% body weight, timothy or alfalfa hay at 1%–1.25% body weight as fresh matter, and small quantities of vitamin and mineral supplements; however, it is likely that the quantity of feed provided would differ somewhat between the stables. There was no pasture at either Training Centre. Because training and general animal care were performed by stable staff not associated with the research team, the feeding practice was not controlled precisely. All horses were transported to the racecourse in a horse trailer on the day before or the day of the race. All racehorses were Thoroughbreds, and their body weights were measured approximately 80 min before post time (i.e., the time the horses entered the starting gate). Body weight was measured at the racecourse using regularly calibrated electronic scales and recorded to the nearest 2 kg. Body weight, age and sex of each horse were recorded. The average body weight and standard error in each month were calculated for time series analysis. The JRA allows horses, which are 2 years of age, to debut in June. Therefore, data were collected after that time. We excluded data for horses ≥8 years of age because of the relatively small sample size of females and geldings. Further, the data for 2-year-old geldings between June and September were not used because of small sample size.

To investigate the seasonal change of body weight and growth as a function of age, we used a seasonal-trend decomposition procedure based on locally weighted regression (STL), which decomposes the time series into seasonal, trend and remainder components [24]. STL is a filtering procedure that iteratively applies locally weighted regression to the observations of the smoothing process in moving time windows, which allows analysis of large numbers of trends and seasonal smoothing [24]. For smoothing parameters, we used the algorithm implemented in the R language. We used STL to investigate seasonal cycles and trends for males, geldings and females, applying it first to all the data and then, in a subgroup analysis, to horses aged 2–4 years and those over 5 years for each sex. These analyses were performed using 2.13.0 R software [25].

## Results

**Fig. 1:**
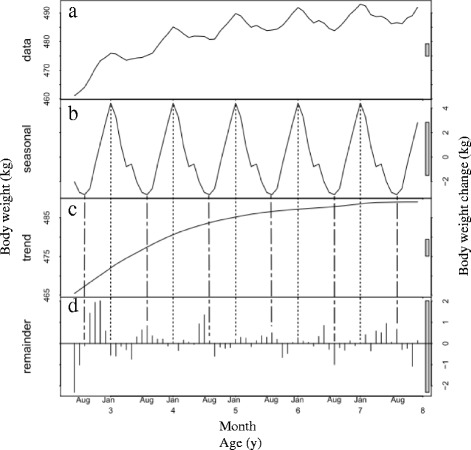
Seasonal-trend decomposition analysis of the body weights of male racehorses. The data are for male horses aged 2–7 years that competed in races held by the Japan Racing Association in the years 2002–2014 (*n* = 377,301). **a** The mean body weight of the horses by age and month. **b** The seasonal component. **c** The trend component. **d** The remainder component after fitting the seasonal and trend components. The dotted vertical lines indicate the month each year when body weight peaked, and the dashed vertical lines indicate the month when the body weight was at its nadir. The grey bars to the right of each panel show the relative scales of the components. Each grey bar represents the same length, but because the plots are on different scales, the bars vary in size

**Fig. 2:**
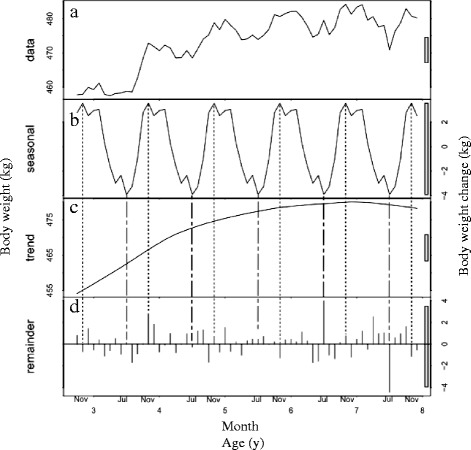
Seasonal-trend decomposition analysis of the body weights of gelding racehorses (*n* = 19,100). **a** The mean body weight of the horses by age and month. **b** The seasonal component. **c** The trend component. **d** The remainder component after fitting the seasonal and trend components. For further details, see the legend for Fig. 1

**Fig. 3:**
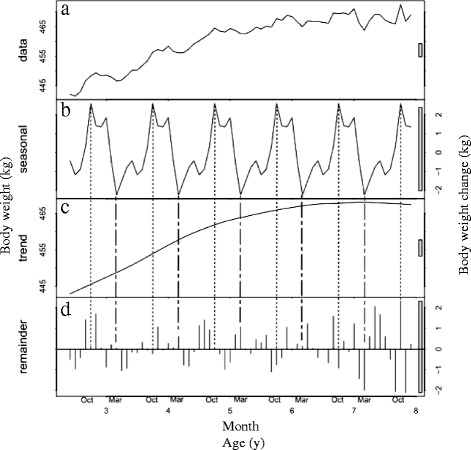
Seasonal-trend decomposition analysis of the body weights of female racehorses (*n* = 236,139). **a** The mean body weight of the horses by age and month. **b** The seasonal component. **c** The trend component. **d** The remainder component after fitting the seasonal and trend components. For further details, see the legend for Fig. 1

We acquired 640,431 body weight measurements for race starts between 2002 and 2014. This included horses that competed in several races (range 1–84 races; median, 6 races). The age of the horses ranged from 2 to 13 years. For STL decomposition, we used 632,540 body-weight measurements of race starts for horses ranging in age from 2 to 7 years, comprising 377,301 males, 19,100 geldings (except for horses 2 years of age from June to September) and 236,139 females. The distribution of age in each month is shown in Table 1. Figs. 1, 2 and 3 show the STL decomposition analyses of body weight for all ages according to sex. The data split by age subgroups is shown in Table 2. Table 3 shows the mean average monthly temperature during the study period at the Miho and Ritto Training Centres.

**Table 1:** Distribution of horses by age and sex competing in Japan Racing Association races, by month

Age (y)	Sex	January	February	March	April	May	June	July	August	September	October	November	December
2	Male						1480	4378	6067	7037	9303	12,702	13,493
	Gelding						(14) ^a^	(40)^a^	(51)^a^	(53)^a^	102	174	218
	Female						1121	3218	4632	5160	6436	8816	9283
3	Male	14,801	13,914	17,045	16,342	17,386	14,659	13,957	13,166	11,475	8600	6948	6797
	Gelding	281	291	435	435	551	505	553	605	493	333	225	273
	Female	10,311	9729	12,636	12,262	13,059	11,139	11,905	12,043	10,003	5860	4194	3764
4	Male	6699	5893	7353	6775	6925	6052	6233	5683	5104	5876	5957	5598
	Gelding	278	279	375	344	375	348	369	374	334	408	399	423
	Female	3471	3144	4025	3702	3957	3430	4117	4044	3308	3421	3303	3089
5	Male	5326	4725	5623	4872	5078	4033	3811	3250	3071	3808	3850	3744
	Gelding	362	347	392	394	456	339	324	350	320	397	381	373
	Female	2602	2358	2790	2470	2631	1967	2161	2190	1782	1888	1803	1690
6	Male	3299	2889	3219	2857	2914	2211	2218	1857	1539	1939	2036	1936
	Gelding	289	260	324	276	293	256	271	253	205	253	230	255
	Female	1309	1159	1193	796	752	573	632	618	536	554	510	451
7	Male	1635	1423	1548	1366	1391	1081	1019	853	654	854	883	791
	Gelding	187	151	212	162	157	136	133	118	97	122	129	116
	Female	339	261	271	194	191	136	171	150	101	118	113	97

^a^The data for 2-year-old geldings between June and September were not used for the seasonal-trend decomposition analysis because of the small sample size

**Table 2:** The months corresponding to the peaks and nadirs of body weight, by age subgroup

		Peak	Nadir
Male	All ages	January	August
	2–4 y	January	August
	≥5 y	January	August
Gelding	All ages	November	July
	2–4 y	October	June
	≥5 y	December	June
Female	All ages	October	March
	2–4 y	October	April
	≥5 y	September	February

**Table 3:** The average monthly temperatures (°C) at the Miho and Ritto Training Centres (mean ± SEM values for 2002–2014)

	January	February	March	April	May	June	July	August	September	October	November	December
Miho	3.8 ± 0.3	4.8 ± 0.3	8.2 ± 0.3	13.0 ± 0.3	17.5 ± 0.2	21.1 ± 0.2	24.8 ± 0.5	26.4 ± 0.3	22.9 ± 0.3	17.4 ± 0.2	11.6 ± 0.3	6.1 ± 0.4
Ritto	3.8 ± 0.2	4.6 ± 0.3	7.5 ± 0.3	13.0 ± 0.3	18.0 ± 0.1	23.2 ± 0.8	26.3 ± 0.4	27.4 ± 0.3	23.7 ± 0.3	17.6 ± 0.3	11.6 ± 0.3	6.3 ± 0.3

The mean body weight of the horses increased from the time of their debuts, with seasonal fluctuations, in all sexes. Over the course of their athletic careers, the mean body weights changed as follows: males, from 461 ± 0.7 kg to 493 ± 0.6 kg; geldings, from 458 ± 2.5 kg to 484 ± 1.7 kg; and females, from 442 ± 0.7 kg to 472 ± 2.4 kg (Figs. 1a, 2a and 3a, respectively). For the male horses, the seasonal component shown in Fig. 1b indicates that their body weight increased in winter, peaked in January and decreased in summer to its lowest value in August. The body weight changes in geldings were similar to those of the intact male horses, although they showed broader peaks in autumn and winter. The body weight of the geldings reached its peak in November and decreased to its lowest value in July (Fig. 2b). The seasonal pattern for the female horses differed from those of the males and geldings. The seasonal component shows that the body weight of females increased in autumn and winter, particularly in October, and decreased in spring, particularly in March (Fig. 3b). The magnitudes of the seasonal fluctuations were 7 kg, 8 kg and 6 kg for the males, geldings and females, respectively.

The plots of the STL trend components show that body weight increased approximately linearly from the horse’s debut until nearly 5 years of age in all sexes (Figs. 1c, 2c, 3c). The trend component in all sexes increased by around 20 kg up to the end of 4 years of age, and by a further 5 kg (i.e., an overall 25 kg increase) at the end of 7 years of age.

In males, there was the same seasonal pattern in both younger horses and older horses, which reached its peak in January and nadir in August (Table 2). In geldings, the peak month was October for younger horses and December for older horses and the nadir month was June in both groups (Table 2). In females, the peak months for younger and older horses were October and September, respectively, and the nadir months were April and February (Table 2).

## Discussion

The present study demonstrates that the body weight of a Thoroughbred racehorse that consumes a relatively constant diet varied circannually. The present study shows that a horse’s sex is associated with the seasonal cycle of body weight. Furthermore, although body weight increased up to approximately 7 years of age, most of the increase occurred before the age of approximately 5 years.

Body weight change depends on energy balance, which is defined as the relationship between energy intake and energy expenditure [26, 27]. This applies to horses [28]. The energy intake of Przewalski horses, which were originally wild herbivores of Central Asia, peaks in autumn and reaches its nadir in late winter [4], whereas energy expenditure is particularly low in winter compared with that in spring and summer [5]. The energy balance of Przewalski horses is positive in autumn, indicating that energy intake is higher than expenditure, and is negative in spring, indicating lower energy intake compared with expenditure. The consequence of these seasonal energy balances would lead to a seasonal change of body weight, which is high in autumn and low in spring [3].

In the present study, the body weights of the Thoroughbred male and gelding racehorses that received sufficient annual nutrition showed seasonal variations that peaked in autumn and winter and reached their nadir in summer. Unlike the Przewalski horses, the racehorses should have received a constant amount of food throughout the year. However, a limitation of the present study is its observational design. Although it is recommended that racehorses should be fed diets according to the results of the equation described in the Methods section, the racehorses studied here were managed by individual stable staff and so we lacked detailed information of the actual energy intake of each horse during the study period. Although establishing the detailed energy intake information by precise feeding control would be ideal, investigating a large sample size of racehorses throughout their athletic career would be difficult. Indeed, because this study analyzed data for a very large number of horses over a period of 13 years, any local or short-term variations in feeding would be expected to be “averaged out” and unlikely to result in noticeable effects. To produce a systematic seasonable effect as we observed would require a consistent, widespread seasonable variation in feed provision that lasted throughout the study period. However, we are unaware of any stable staff changing the feeding protocol according to the season (personal communication). In addition, the annual fluctuation in the body weight of the horses in this study was within 8 kg, which is equivalent to less than 2% of their mean body weight. This compares with 22 kg fluctuations in the annual mean amplitude of the body weight of Przewalski horses in a semi-natural condition, equivalent to 7% of their mean body weight [3]. This suggests that the horses in the present study were fed more consistently than horses housed under semi-natural conditions.

Thus, the results of this study suggest that male and gelding racehorses were energetically abundant in autumn and winter, and deficient in summer. The assumption that there was little change in energy intake suggest that these results are due to the seasonal changes in energy expenditure (high in summer and low in winter); this is consistent with a previous report [8].

Seasonal environmental factors such as temperature or photoperiod may explain the seasonal changes in metabolic rate. The range in average monthly temperature over the year at both training centres was about 23 °C, with the highest temperatures in August and the lowest in January. The horses were stabled at the ambient temperature, so the high temperatures in summer may have increased their metabolic rate and the low temperatures in winter may have reduced it, which would be consistent with the results of previous studies [8, 29, 30]. Although a long photoperiod induces a higher metabolic rate than a shorter photoperiod in rats and cats [9, 31], there are no published studies, to our knowledge, on the direct relationship between photoperiod and energy expenditure by horses. Further studies are required to confirm this potential relationship.

In contrast to male and gelding horses, the body weight of female horses peaked in autumn and reached the nadir in spring, consistent with a previous report [11]. These findings suggest that female horses employ a mechanism that maintains energy balance that differs from that of male and gelding horses. The sexual cycle of female horses may provide an explanation. For example, the locomotor activity of female Thoroughbred horses is higher during breeding season [32]. The metabolic rate can be higher in breeding season, similar to behavioural changes, because Thoroughbred racehorses stabled in the training centres maintain their oestrous cycle during training [33]. Further studies are required to identify sex-specific mechanisms that regulate body weight.

The seasonal fluctuations in body weight may have important implications for equine clinicians, racehorse trainers, or both. Our previous study shows that racehorses that are heavy at race time are at higher risk of superficial digital flexor tendon injury compared with horses that weigh less [20]. Further, increases in body weight might affect the results of the submaximal exercise test [21]. Therefore, equine clinicians and racehorse staff should adjust the amount of feed according to seasonal body weight changes such as the reduction of feeding amount in autumn and winter in males and geldings, and in autumn in females.

We show here that the body weight of Thoroughbred racehorses increased 25 to 30 kg during their athletic career. According to the trend component, most of the increase was observed until horses approaches approximately 5 years of age. To our knowledge, this is the first report that determined the time interval associated with the increase in body weight of Thoroughbred racehorses during their athletic career. This finding is reasonable because the last epiphysis closure in a cervical vertebra occurs at the age of 4–5 years [22], indicating that horses mature by the end of their fourth year. Moreover, racing performance or average racing speed peaks at approximately 4.5–5 years of age [34, 35]. Together, the present and previous studies indicate that the physiological development of Thoroughbred racehorses is complete just before 5 years of age. It would be interesting to investigate the association between body weight increase and anatomical development, such as the development of bones and joints. However, this would be difficult to achieve for a large sample size. The mean body weights of the Thoroughbred racehorses at the completion of physiological development were approximately 490 kg, 480 kg and 465 kg for males, geldings and females, respectively. We propose the use of these values as standards for Thoroughbred racehorses in Japan.

The remainder component is an indication of the residual data after subtracting the seasonal and trend components [24] and shows variations due to random features. However, there appeared to be a small element of seasonality in the remainder component for the young male horses, especially those aged 2–3 years (Fig. 1d). Another age-related factor was that there were fewer older horses than younger ones, especially among the females (Table 1). It is possible that different number between older and younger horses may have had different seasonal patterns according to ages. For these reasons, we divided the data into two groups for each sex, younger horses (2–4 years) and older horses (those over 5 years). However, the results showed almost the same seasonal pattern as the undivided datasets, with the body weight of the males and geldings reaching a peak in autumn and winter, and their nadir in summer whereas the body weight of the females peaked in autumn and was at its lowest in spring, although the divided data showed a slightly wider range than the combined data for all ages. These additional results suggest that the datasets were appropriately divided into seasonal, trend and remainder components.

We should take care to correctly interpret the data acquired from the analysis of body weight change that occurs each month or season. For example, overall average body weight in JRA may be lower in June compared with May, because 2-year-old horses with lighter body weights compared with older horses are admitted to the JRA in June. To avoid such a misleading analysis, we applied STL techniques to body weight data classified according to age, which is a useful technique in diverse disciplines [36, 37]. The STL technique is an effective tool for visualizing and clarifying time series events by dividing them into seasonal, trend and remainder components [24]. We used this technique here to identify the seasonal change associated with growth.

Due to the JRA system described in the Introduction section, the distribution of age and month relatively changed according to the season. Especially after August, the number of 2-year-old horses increased while the number of 3-year-old horses decreased. If there were seasons specific for any age or gender, racing system can have possible role on body weight change. For example, if autumn and winter season were for only 2-year-old horses, reduction of workload for over 3-year-old horses might cause overall increase of body weight. However, races from Maiden to Group One are held every week regardless of age and gender in JRA. Therefore, we speculate that JRA system did not affect our interpretation.

## Conclusions

The present study shows that Thoroughbred racehorses exhibited annual rhythms of body weight, suggesting that they maintain a seasonal energy balance. Further, there was a sex difference in the seasonal pattern of body weight changes, that is, the body weights of the male horses and geldings peaked in autumn and winter and reached their nadir in summer whereas the body weight of the female horses peaked in autumn and reached its nadir in spring. Additionally, most of the increase of body weight of Thoroughbred racehorses during their athletic careers is completed just before the age of 5 years. These results should be useful for optimizing the nutritional management of athletic horses.
